# Antibodies Induced by Homologous or Heterologous Inactivated (CoronaVac/BBIBP-CorV) and Recombinant Protein Subunit Vaccines (ZF2001) Dramatically Enhanced Inhibitory Abilities against B.1.351, B.1.617.2, and B.1.1.529 Variants

**DOI:** 10.3390/vaccines10122110

**Published:** 2022-12-09

**Authors:** Xuesong Xu, Yi Hong, Erjing Chen, Yaping Wang, Biao Ma, Jiali Li, Wei Su, Yuxin Zhou, Mingzhou Zhang

**Affiliations:** 1 Zhejiang Provincial Key Laboratory of Biometrology and Inspection & Quarantine, China Jiliang University, Hangzhou 310018, China; 2 Hangzhou Quickgene Sci-Tech. Co., Ltd., Hangzhou 310018, China; 3 Wenzhou MeiZhong Medical Laboratory, Wenzhou 325000, China; 4 College of Life Science, China Jiliang University, Hangzhou 310018, China

**Keywords:** homologous, heterologous, inactivated vaccine, recombinant protein subunit vaccine, inhibitory abilities

## Abstract

Safe and effective vaccines for Corona Virus Disease 2019 (COVID-19) can prevent the virus from infecting human populations and treat patients infected with severe acute respiratory syndrome coronavirus 2 (SARS-CoV-2). In this study, we discuss the inhibitory abilities of primary and booster vaccine-induced antibodies inhibitory ability toward the SARS-CoV-2 wild-type strain, as well as B.1.1.7, B.1.351, P.1, B.1.617.2, and B.1.1.529. We confirmed these antibodies had the strongest inhibitory effects on the wild-type strain and cross-inhibition activities against other mutant strains after two inactivated vaccine doses. However, the B.1.351, B.1.617.2 and B.1.1.529 mutants exhibit antibody resistance in the vaccine serum. Antibodies induced by homologous inactivated vaccines (n = 92) presented more effective inhibition against tested SARS-CoV-2 strains (*p* < 0.0001), especially B.1.351, B.1.617.2, and B.1.1.529 mutant strains, which had strong immune escape characteristics. In addition, a heterologous booster vaccination (n = 50) of a protein subunit vaccine ZifiVax (ZF2001) significantly restored humoral immune responses and even showed an increasing response against wild-type, B.1.351, B.1.617.2, and B.1.1.529 than homologous inactivated vaccines. Our analysis of the humoral immune response elicited by the different vaccine regimens, including inhibiting antibodies, indicated that a booster, whether homologous or heterologous, could be essential for achieving greater efficacy against SARS-CoV-2.

## 1. Introduction

The Corona Virus Disease 2019 (COVID-19) outbreak, caused by severe acute respiratory syndrome coronavirus 2 (SARS-CoV-2) infection, began in December 2019 and immediately threatened the people’s lives worldwide. As of 16 November 2022, a total of 632,953,782 confirmed cases of COVID-19, including 6,593,715 deaths, were reported globally. As of 8 November 2022, upwards of 12,885,748,541 vaccine doses were used for inoculation [1].

The receptor-binding domain (RBD) of the spike protein, which exhibits immunodominance [2,3], was a predominant target for both natural infection and vaccine-induced antibodies [4]. Specific inhibitory antibodies produced by natural infection or vaccine can bind to the spike protein, blocking the formation of the SARS-CoV-2-RBD-human angiotensin-converting enzyme 2 (hACE2) complex and cutting off the main route of infection. Studies have found that inhibitory antibodies targeting spike proteins are of great significance for preventing and treating COVID-19. Under the influence of the selective pressure of antiviral drugs, specific monoclonal antibodies, and other therapeutic measures, SARS-CoV-2 mutations continuously evolve with genome replication, and mutation rates have been accelerating rapidly [5]. Mutant strains such as Alpha (B.1.1.7, Britain), Beta (B.1.351, South Africa), Gamma (P.1, Brazil), Delta (B.1.617.2, India), and Omicron (B.1.1.529, Botswana), exhibit mutations that lead to increasing heritability and immune evasion. The emergence of mutant strains raises significant concerns about viral transmissibility, disease severity, and reinfection rates, impacting the effectiveness of monoclonal antibodies targeting RBD and polyclonal antibodies elicited by infection or vaccination [5,6].

The emergence of mutant strains poses a continuing threat to global public health. Vaccines must be able to develop a herd immunity barrier against SARS-CoV-2 infection. Anti-SARS-CoV-2 vaccines can be classified as inactivated vaccines (CoronaVac, BBIBP-CorV), mRNA vaccines (mRNA-1273, BNT162b1), viral vector vaccines (AZD1222-Vaxzevria, Ad26.COV2. S), and recombinant protein subunit vaccines (NVX-CoV2373, ZF-UZ-VAC-2001), etc. These vaccines elicit excellent neutralizing activity against the SARS-CoV-2 wild-type strain at the prescribed doses [7,8,9,10]. However, with the emergence of circulating mutant strains of SARS-CoV-2, the protective capacity of the vaccines has gradually weakened or been lost altogether [11,12,13,14,15].

ZifiVax (ZF2001, Anhui Zhifei Longcom Biopharmaceutical Co., Ltd., Anhui, China) is a recombinant protein subunit vaccine whose antigen is a dimeric form of the receptor-binding domain (RBD) [16]. This antigen, which encodes SARS-CoV-2 RBD (residues 319–537), is manufactured in Chinese hamster ovary cells [17]. The three-dose ZF2001 vaccine regimen was more than 75% effective in preventing severe COVID-19 disease and exceeded the preferred WHO criteria for the target product configuration of COVID-19 vaccines (≥70%) [18]. ZF2001 exhibited cross-protection against B.1.1.7 at 92.7–88.3% and B.1.617.2 at 81.4–76.1%, respectively [18]. In addition, it contributed to higher neutralization levels of omicron subvariants [19]. The National Health Commission of the China deployed a sequential immunization booster. Members of the target population who have received the BBIBP-CorV and CoronaVac inactivated vaccines can also choose the recombinant protein subunit vaccine ZF2001 or the adenovirus vector vaccine Ad5-nCoV (CanSino Biologics Inc., Tianjin, China) for sequential booster immunization [20]. ZF2001 was registered in Uzbekistan [21], approved for emergency use in Indonesia and Colombia [22], and approved as a sequential (heterologous) booster shot in Indonesia.

We performed an vitro experiment binding the antibodies with the SARS-CoV-2 spike protein RBD to block the RBD protein complex from forming with the ACE2 receptor. The detection of antibodies by blocking RBD-hACE2 binding has been positively correlated with neutralizing serum antibody titers [4]. Our research provided a comprehensive evaluation of serum antibodies induced by inactivated vaccines (CoronaVac/BBIBP-CorV) followed longitudinally for up to six months. At the same time, the degree of immune escape from each epidemic mutant was observed, especially B.1.351, B.1.617.2 and B.1.1.529. Our study also demonstrated enhancement in the cross-inhibiting activity of immune serum elicited by the homologous inactivated and heterologous vaccines, suggesting continued efficacy against emerging variants and the benefits of booster vaccinations.

## 2. Materials and Methods

### 2.1. Sample Collection

Serum samples were collected and classified according to the time point of serum collection. The time points and information regarding the serum samples are displayed in Table 1.

In addition, we regularly collected samples from 32 volunteers at the following time points: day 14 and 21 following the first dose, day 7, 14, 21, 28, 58, 88, 118, and 180 after the second dose, and day 7 after the homologous CoronaVac booster. The 32 volunteers who received CoronaVac had neither SARS-CoV-2 infection history nor SARS-CoV-2 inhibiting antibodies prior to vaccination. All the vaccinated participants provided informed consent prior to data and specimen collection.

Serum samples were collected in anticoagulant tubes and centrifuged at 5000 rpm for 10 min (Thermo Fisher Scientific, Waltham, MA, USA). We stored the samples in a laboratory serum biobank at −20 °C. On the day of detection, the frozen sample was thawed at 4 °C for one hour. The rethawed samples were subjected to eddy current treatment prior to analysis. The research program was consistent with the Declaration of Helsinki.

### 2.2. Cell Culture, Expression and Purification of SARS-CoV-2 Strains RBD, and hACE2 Recombinant Proteins

Expi293F cells (Thermo Fisher Scientific, Waltham, MA, USA) were cultured in suspension in Expi293F expression medium (Thermo Fisher Scientific, Waltham, MA, USA) at 37 °C with 8% CO_2_. Expi293F is a highly transfectable cell line that transiently expresses proteins. Cells are either grown in batches or supplemented in medium depending on the number of cells growing and the viability of transfected cells ≥98%. Recombinant plasmids with DNA sequences encoding the spike proteins RBD of the SARS-CoV-2 wild-type and mutant strains (B.1.1.7, B.1.351, P.1, B.1.617.2, B.1.1.529) from Hangzhou Quickgene Sci-Tech. Co., Ltd. (Hangzhou, China) were transfected into Expi293F cells to obtain SARS-CoV-2 strains RBD recombination proteins, using the ExpiFectamine™ 293 transfection kit (Thermo Fisher Scientific, Waltham, MA, USA). The same procedure was used for hACE2 protein expression. The cell suspension was centrifuged at 10,000 rpm for 20 min in a refrigerated centrifuge and filtered through a 0.22 µm filter to obtain the supernatant on the seventh day after transfection. Figure S1 shows information for purified hACE2 and RBD proteins.

### 2.3. Enzyme-Linked Immunoassay of Competition

An enzyme-linked immunoassay of competition was set up to investigate the inhibition of serum antibodies. First, 96-well microplates (Costar, Kennebunk, ME, USA) were precoated with hACE2 protein at 60 ng/well in 100 µL of 50 mM carbonate-bicarbonate coating buffer (pH 9.85) overnight at 4 °C for 24 h. Then, they were blocked with a blocking buffer at 4 °C for 48 h. After adding serum samples which was diluted into 50 µL of sample buffer to the hACE2-coated plates, wild-type-RBD-HRP diluted into 50 µL of enzyme buffer at optimal concentration was added into plates to detect antibodies inhibiting the ability of wild-type strain. We selected the corresponding RBD-HRP and optimal concentration according to different mutant strains to evaluate the antibody inhibition ability. We set the reaction system for 30 min at 37 °C. Unbound HRP-conjugated RBD was removed using three washes with phosphate-buffered saline, 0.05% Tween-20 (PBST). Then, 100 µL of tetramethylbenzidine (TMB, Invitrogen, Waltham, MA, USA) was added to the plates for 15 min in the dark at 37 °C. We added TMB stop solution to stop the reaction and used a microplate reader (Thermo Fisher Scientific, Waltham, MA, USA) to record the absorbance readings at 450 nm. This method was a suppression assay whose color intensity was inversely proportional to the level of inhibitory antibodies in the samples. The data were interpreted by calculating the percentage of RBD-HRP binding inhibition. The optimal concentration of RBD-HRP was determined according to the standard of negative control, and the OD value was 2.0 ± 0.2. Inhibition (%) = (1 − OD_sample_/OD_negative control_) × 100. Our enzyme-linked immunoassay of competition test determined a negative cut-off value at 30% inhibition from testing over negative (n = 89) human serum (Figure S2). The negative cut-off value was similar to other reported studies [23,24]. A positive serum was considered if the inhibition rate was ≥30%. Antibody inhibition rates of 70–100% were considered high, 50–70% were considered moderate, and 30–50% were considered low.

### 2.4. Detailed Studies on the Primary Inactivated Vaccine-Induced Inhibitory Antibodies Cross-Inhibition against Wild-Type and B.1.1.7, B.1.351, P.1, B.1.617.2, and B.1.1.529 Mutant Strains

We used the average inhibition rate to evaluate the ability of vaccine serum antibodies to inhibit the hACE2 protein from binding with the spike protein RBD of each SARS-CoV-2 strain. We researched vaccine-induced antibodies at four time points following the primary dose of inactivated vaccine (CoronaVac/BBIBP-CorV), inhibiting the RBD of SARS-CoV-2 wild-type strain or mutant strains (B.1.1.7, B.1.351, P.1, B.1.617.2, and B.1.1.529) from binding with hACE2. The four time points included the third week (n = 68) following the first inactivated vaccination dose and the first (n = 201), third (n = 127), and fifth (n = 68) months following the second dose. Based on the comparison between groups, we study the cross-inhibition ability of antibodies in the serum of inactivated vaccines (CoronaVac/BBIBP-CorV) against wild-type and mutant strains of SARS-CoV-2.

### 2.5. Longitudinal Kinetics and Duration of Inactivated Vaccine-Induced Inhibitory Antibodies

In addition, serum samples from 32 individuals who received two complete doses of CoronaVac were evaluated in detail at 10 time points. We analyzed the average inhibition rates of serum against the wild-type and mutant strains at each time point and trends in antibodies’ inhibition rates. We used our results to support research on the longitudinal kinetics and effective duration inhibiting antibodies in the serum. We also analyzed the rate of positive antibodies on day 180 following the second dose of vaccine.

### 2.6. Evaluation of Antibodies Inhibitory Ability with Inactivated Homologous and Inactivated/Recombinant Protein Subunit Vaccine Prime-Boost Heterologous Vaccination

Twenty out of thirty-two volunteers received a single CoronaVac booster 6 months after their primary vaccination. By comparing the inhibition rates of serum antibodies on day 7 after the second does with the same time point of the booster, we determined whether the inhibitory effect on wild-type, B.1.1.7, B.1.351, P.1, B.1.617.2, and B.1.1.529 increased significantly after the booster.

**Figure 1:**
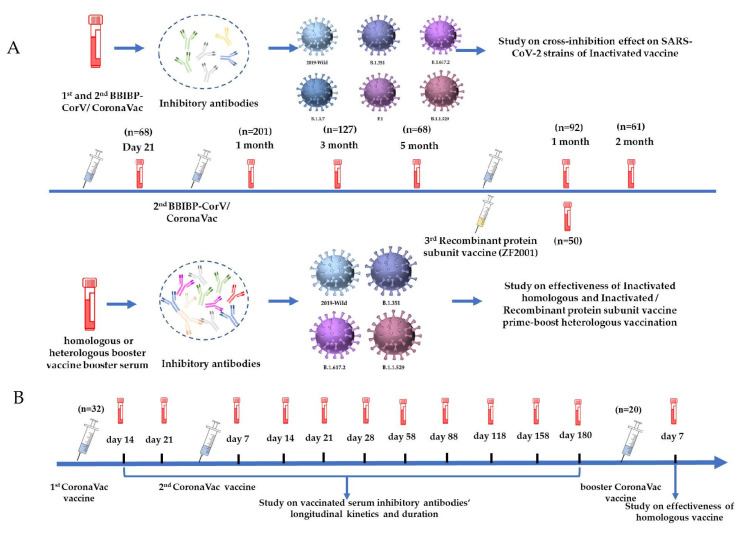
Collection of vaccine serum samples and study of antibody vaccine-induced effectiveness on SARS-CoV-2 strains. (**A**) Evaluation antibodies of serum samples collected at the 4 time points: the 3rd week (n = 68) following the first inactivated vaccination dose and the 1st month (n = 201), 3rd month (n = 127), and 5th month (n = 68) following the second dose of inactivated vaccine (CoronaVac/BBIBP-CorV) and serum samples from the first (n = 92) and second months (n = 61) after a homologous booster and from the first month (n = 50) following recombinant protein subunit vaccine (ZF2001) as a heterologous booster. (**B**) Evaluation of serum sample antibodies from 32 individuals who received two complete doses of CoronaVac at 10 time points.

The inhibitory abilities of serum antibodies against the wild strain, B.1.351, B.1.617.2, and B.1.1.529, after homologous and heterologous boosters were evaluated to investigate booster effectiveness. The samples studied involved serum samples from the first (n = 92) and second months (n = 61) after a homologous booster with the inactivated vaccine (CoronaVac/BBIBP-CorV) and from the first month (n = 50) following recombinant protein subunit vaccine (ZF2001) as a heterologous booster after inactivated vaccine-based injection. In addition, serum before receiving the booster vaccine was collected from 54 out of 92 volunteers who received a homologous booster vaccine. Our research methods and strategies are shown in Figure 1.

### 2.7. Data Statistics

We used GraphPad Prism 8.0.2 software (San Diego, CA, USA) for data visualization. The *p* value was expressed in terms related to the alpha value. The significance value for all analyses was set to 0.05, and 0.01 was considered an extremely significant value. Brown–Forsythe, Welch ANOVA, and Games–Howell multiple comparison tests were used to determine the statistical significance between groups. A paired *t*-test was used to analyze the differences in serum antibodies between the seventh day following the primary two vaccinations and booster.

## 3. Results

### 3.1. Inhibitory Antibody Induced by CoronaVac/BBIBP-CorV Exhibited the Highest Inhibition Capacity against SARS-CoV-2 Wild-Type and Cross-Inhibiting Activities against SARS-CoV-2 Mutant Strains

The average inhibition rates of antibody induced by CoronaVac/BBIBP-CorV against wild-type and mutant strains were analyzed at four time points. The number of positive serum samples for wild-type strain: 36/68, B.1.1.7: 42/68, B.1.351: 11/68, P.1: 6/68, B.1.617.2: 34/68, and B.1.1.529: 18/68 at the third week following the first dose. The average antibody inhibition rates of serum collected at the third week after the first dose (n = 68) and at the first (n = 201), third (n = 127) and fifth (n = 68) months after the second dose of inactivated vaccine to wild-type, B.1.1.7, B.1.351, P.1, B.1.617.2, and B.1.1.529 are shown in Table 2.

The differences in inhibition rates of serum antibodies against each strain are shown in Table S1. The average inhibition rates of serum antibodies against wild-type, B.1.1.7, B.1.351, P.1, B.1.617.2, and B.1.1.529 were 82.17%, 75.00% (*p* < 0.0001), 65.04% (*p* < 0.0001), 69.99% (*p* < 0.0001), 58.97% (*p* < 0.0001), and 54.79% (*p* < 0.0001) after the first month of the second dose, respectively. Inactivated vaccines produced high-titer specific inhibitory antibodies targeting SARS-CoV-2 spike proteins. Inhibitory antibodies induced by CoronaVac/BBIBP-CorV exhibited the highest inhibition capacity against the SARS-CoV-2 wild-type and cross-inhibiting activities against SARS-CoV-2 mutant strains.

**Figure 2:**
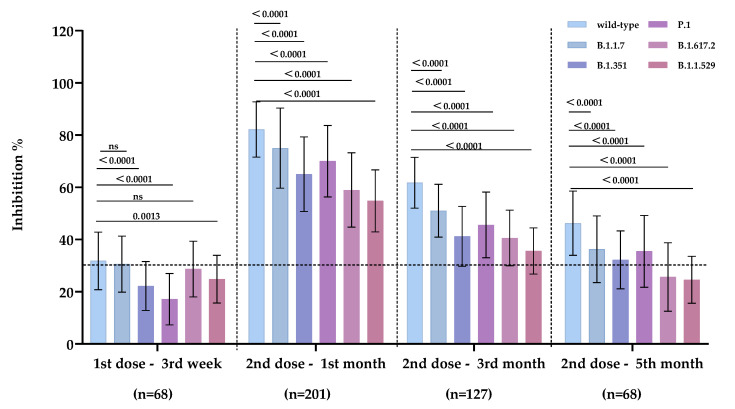
Antibodies induced by the primary inactivated vaccine exhibited inhibitory activity against wild-type, B.1.1.7, B.1.351, P.1, B.1.617.2 and B.1.1.529. Detection time points: 3rd week after the first dose (n = 68), 1st month (n = 201), 3rd month (n = 127), and 5th month (n = 68) after the second dose of inactivated vaccine. The dotted lines represent the cutoff at 30% inhibition. ns: no significance.

In the first month after the second dose, the average inhibition rates against B.1.351, B.1.617.2, and B.1.1.529 mutants decreased by 17.12% (CI: 13.53–20.71%), 23.20% (CI: 19.61–26.78%), and 27.37% (CI: 24.16–30.59%), with the wild-type strain as the control. Additionally, B.1.351, B.1.617.2, B.1.1.529 mutants decreased by 20.55% (CI: 16.71–24.39%), 21.15% (CI: 17.48–24.83%), 26.15% (CI: 22.80–29.50%), in the third month, respectively. The antibodies’ inhibition abilities were significantly reduced in the mutant strains B.1.351, B.1.617.2, and B.1.1.529, with high level against the wild-type strain. The average inhibition rates of serum antibodies against wild-type, B.1.1.7, B.1.351, P.1, B.1.617.2 and B.1.1.529 were 46.25%, 36.28%, 32.23%, 35.48%, 25.64%, and 24.58% during the fifth month after the second dose. The average inhibition of serum antibodies decreased to a limited cross-reactivity level for wild-type, B.1.1.7, B.1.351, and P.1 after five months. At the same time, it become undetectable for B.1.617.2 and B.1.1.529 (Figure 2).

Serum inhibition curves and IC50s were generated from five volunteers (n = 5) who received inactivated vaccines (Figure S4 and Table S4) to evaluate the inhibitory activity of antibodies elicited by the two primary inactivated vaccines (CoronaVac/BBIBP-CorV).

### 3.2. Longitudinal Kinetics and Duration Effectiveness of Vaccinated Serum Inhibiting Antibodies

**Figure 3:**
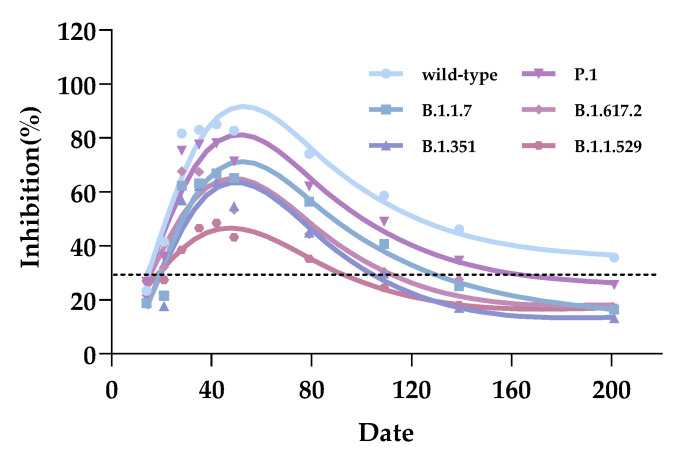
Longitudinal kinetics and duration effectiveness of vaccinated serum inhibiting antibodies. The ordinate of the coordinate axis is the inhibition rates, and the abscissa represents the different sampling dates following receipt of the first CoronaVac dose. The range of 28–35 days received the second dose of CoronaVac. The dotted lines represent the cutoff at 30% inhibition.

To determine the persistence of inactivated vaccine antibodies’ inhibitory capacity, we followed 32 volunteers for 6 months after receiving two doses of CoronaVac vaccine. Serum samples were collected at certain intervals. The average inhibition rates of serum samples collected against wild-type and mutant strains are shown in Table S2 and Figure S3. At two time points following the first dose (Figure S3A,B), the average inhibition rates of serum antibodies against each SARS-CoV-2 strain were close to the 30% negative cut-off value. The inhibitory trend of specific antibodies is exhibited in the data analyses and kinetic image (Figure 3). The inhibitory abilities of serum antibodies against all targeted strains (wild-type, B.1.1.7, B.1.351, P.1 and B.1.617.2: *p* < 0.0001; B.1.1.529: *p* = 0.0031) sharply increased and reached their peak within 3 weeks following the second dose. The average inhibition rates of serum antibodies against SARS-CoV-2 wild-type, B.1.1.7, B.1.351, P.1, B.1.617.2, and B.1.1.529 were 82.74%, 65.21%, 54.67%, 71.38%, 53.42%, and 43.26% at day 28 following the second dose, respectively. For wild-type, B.1.1.7, P.1, and B.1.1.529 strains, antibody inhibition rates lasted for about 60 days; however, the inhibitory level differed between the four strains. Subsequently, antibody inhibition rates were on the decline [25]. We also measured the effectiveness of serum samples blocking the binding hACE2 with each strain of RBD in the sixth month following the second dose. At day 180, against wild-type, B.1.1.7, B.1.351, P.1, B.1.617.2, and B.1.1.529 strains, the proportions of detectable positive serum samples were 71.86% (23/32), 15.63% (5/32), 3.13% (1/32), 40.63% (13/32), 12.50% (4/32), and 15.63% (5/32), respectively. The average inhibition rate against the wild-type strain was 35.71%, higher than the negative cutoff threshold. It was speculated that the detectable positive antibody against the wild-type strain lasted more than 6 months [26].

### 3.3. Vaccinated Serum Antibodies Showed Extensive Inhibitory Activities following Homologous Booster Vaccination

**Figure 4:**
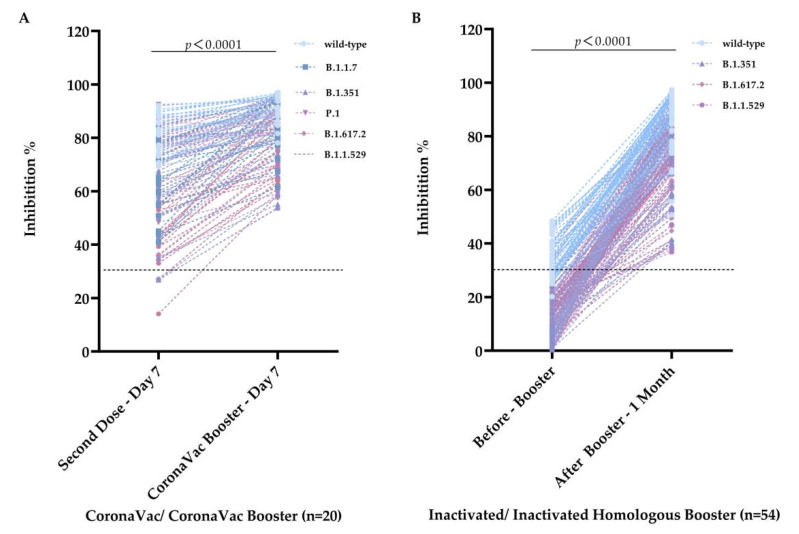
Comparison of the inhibition rates between antibodies induced pre- and post-booster. (**A**) Variation of inhibition rates on 7th day from after the second dose of the CoronaVac booster. (**B**) Variation of inhibition rates from pre- to post-receiving homologous booster. The dotted lines represent the cutoff at 30% inhibition.

Twenty of the thirty-two volunteers who received a single CoronaVac booster and inhibitory antibodies on day 7 were directly compared for receiving two primary vaccine doses to the booster. A paired *t*-test was performed on the collected data (Figure 4A).

On the 7th day after receiving the CoronaVac booster, the inhibition rates against wild-type, B.1.1.7, B.1.351, P.1, B.1.617.2 and B.1.1.529 were 90.47%, 85.15%, 79.41%, 85.55%, 80.93%, and 74.36%, respectively. Our results showed that average inhibitory rates against the wild-type, B.1.1.7, B.1.351, P.1, B.1.617.2, and B.1.1.529 increased by 8.93% (CI: 4.26–13.59%), 21.55% (CI: 14.03–29.07%), 22.00% (CI: 12.78–31.22%), 12.03% (CI: 4.51–19.54%), 14.74% (CI: 5.12–24.28%) and 32.60% (CI:25.64–39.55%). The homologous booster significantly increased antibodies’ inhibitory activities for tested strains and enhanced the extensive cross-inhibition of SARS-CoV-2 mutant strains. It also had a high inhibition level of 90% against the wild-type strain. Meanwhile, average inhibition increased significantly with the booster, especially against B.1.1.529.

In addition, serum samples before receiving the booster vaccine were collected from 54 out of 92 volunteers who received the homologous booster vaccine. The inhibition rates of serum antibodies against wild-type, B.1.351, B.1.617.2, and B.1.1.529 before and after homologous booster vaccination were compared to determine the change in humoral immune response after booster vaccination (Figure 4B). The average inhibition rates against wild-type, B.1.351, B.1.617.2, and B.1.1.529 increased by 52.26% (CI: 48.81–55.71%), 64.89% (CI: 60.36–69.42%), 61.01% (CI: 56.92–65.11%), and 58.06% (CI: 53.56–62.57%), respectively. These results showed that the homologous booster immediately restored the immune response and promoted more antibodies.

### 3.4. Heterologous Vaccination with the Recombinant Protein Subunit Vaccine Significantly Recalled and Increased the Humoral Immune Responses against Wild-Type, B.1.351, B.1.617.2, and B.1.1.529 Mutant Strains

**Figure 5:**
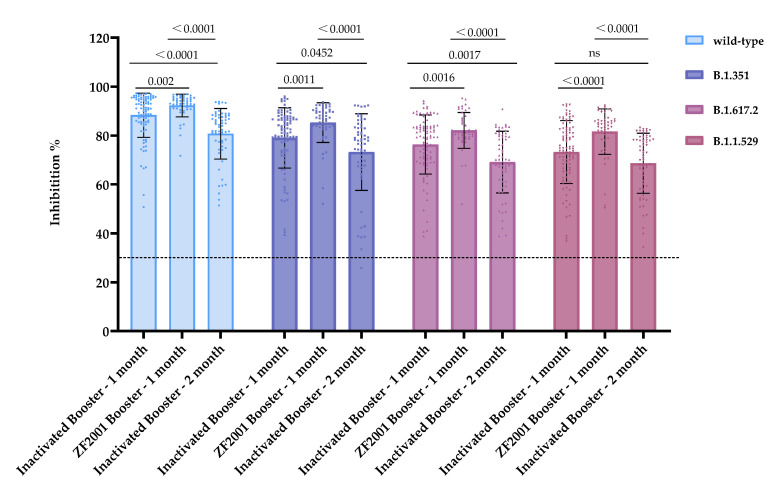
Comparison between the antibody inhibition rates of homologous and heterologous boosters against wild-type, B.1.351, B.1.617.2, and B.1.1.529. The dotted lines represent the cutoff at 30% inhibition. ns: no significance.

We aimed to investigate the effectiveness of the recombinant protein subunit vaccine (ZF2001) as a heterologous booster for inhibiting hACE2 from binding with RBD of wild-type, B.1.351, B.1.617.2, and B.1.529 strains. We collected homologous inactivated/inactivated prime-boost individuals’ serum samples in the first (n = 92) and second (n = 61) months and inactivated/recombination protein submit vaccine (ZF2001) prime-boost samples (n = 50). The inhibitory abilities of antibodies against wild-type, B.1.351, B.1.617.2, and B.1.1.529 are evaluated in Table 3 and Figure 5. The difference between the inhibition rates of each booster vaccine among wild-type, B.1.351, B.1.617.2, and B.1.1.529 are shown in Table S3.

The ZF2001 booster vaccine had an estimated 85.31% inhibition rate against B.1.351, 82.10% against B.1.617.2, and 81.27% against B.1.1.529. The inhibition rates of the ZF2001 booster serum on wild-type (*p* = 0.002), B.1.351 (*p* = 0.0011), B.1.617.2 (*p* = 0.0016), and B.1.1.529 (*p* < 0.0001) also increased with respect to the homologous booster. Both homologous and heterologous booster vaccination exhibited the highest efficacy against the wild-type strain and showed extensive cross-inhibition of B.1.351, B.1.617.2, and B.1.1.529. Although the inhibitory effects of the serum following the booster vaccine on the B.1.351, B.1.617.2, and B.1.1.529 fell slightly, most of the inhibitory activity remained.

The preliminary results indicated inhibition rate decreases of 7.56% (*p* < 0.001), 5.77% (*p* = 0.0452), 7.22% (*p* = 0.0017), and 0.53% (*p* = 0.9558) in the second month against wild-type, B.1.351, B.1.617.2, and B.1.1.529, respectively, compared with the first month following the homologous booster. The inhibitory abilities of the serum antibodies in the second month following the homologous inactivated vaccine against wild-type, B.1.351, B.1.617.2, and B.1.529 were ≥69.12%. In addition, to evaluate the inhibiting activity of antibodies, we generated serum inhibition curves and IC50s from volunteers (n = 5) who received the inactivated homologous and heterologous (ZF2001) boosters for wild-type, B.1.351, B.1.617.2, and B.1.1.529 variants (Figure S5 and Table S5).

Compared with the primary dose, homologous and heterologous booster-induced antibodies enhanced inhibitory abilities against B.1.351, B.1.617.2, and B.1.1.529 mutant strains, which possess strong immune escape ability.

## 4. Discussion

The World Health Organization’s approval of China’s COVID-19 vaccines is essential for keeping the pandemic within limits. CoronaVac is 100% effective at preventing severe disease and death [9]. CoronaVac/BBIBP-CorV vaccines are used in China and about 110 developing countries [27]. Our data have broad applicability and contribute to understanding the potential impact of the primary booster vaccination. CoronaVac/BBIBP-CorV are driving China’s massive internal immunization campaign. The immune response provided by CoronaVac vaccines and the neutralization and therapeutic effects of vaccine-induced antibodies is of great concern. In addition, inactivated vaccines have a theoretical advantage in that they contain whole viruses with nucleoproteins that may promote a broader immune response than other vaccine platforms using only spike proteins [28], thus reducing the escape of variants from vaccine immunity [29]. It is generally accepted that polyclonal antibodies produced by vaccination target multiple epitopes [30]. The design of the vaccine was determined early in the pandemic based on virus sequences first reported in 2019. The effects of multiple mutations of SARS-CoV-2 mutant strains RBD on vaccine-induced immunity are worth studying.

We evaluated and compared antibodies’ ability to inhibit the binding of hACE2 with SARS-CoV-2 strains RBD (wild-type, B.1.1.7, B.1.351, P.1, B.1.617.2, and B.1.1.529) following the reception of inactivated vaccines. We found that antibodies induced by the CoronaVac vaccine with the two primary doses inhibited the binding of hACE2 with SARS-CoV-2 strains. Protection from the first dose up until administration of the second dose was limited [31]. We analyzed the kinetic trend of the antibodies. The inhibitory abilities of serum antibodies against all the targeted strains sharply increased following the second dose and reached their peak within 3 weeks [3]. These trends suggested an extensive range of serum inhibitory activities 3–12 weeks following the second dose of CoronaVac. This range was in accordance with the inhibitory immunity of polyclonal antibodies induced by the BNT162b2 and mRNA-1273 vaccines [32,33,34]. The inhibitory abilities of the antibodies waned six months after the second injection [29]. The antibodies’ inhibition trends were consistent with the serum antibodies of naturally infected patients [25] until the average inhibition rates of the antibodies turned negative.

Antibodies had the strongest inhibitory effects on the wild-type strain and extensive cross-inhibition activities with other mutant strains after receiving a second dose of inactivated vaccine (CoronaVac/BBIBP-CorV). We also noted that serum antibodies could inhibit the B.1.351 and B.1.617.2 mutants to a certain extent. However, this inhibition level could not be sustained for a longer period. Additionally, the antibodies’ ability to inhibit mutant B.1.1.529 was significantly reduced compared to the wild-type and other strains. B.1.1.529 strains exhibited significant inhibitory resistance, similar to B.1.351 and B.1.617.2 [35], which exhibit nearly complete resistance to neutralization by convalescent plasma [30]. This finding is probably related to immune escape caused by the mutations of spike proteins RBD in the three mutant strains [36,37]. N501Y, substituted in two immunodominant regions of the spike in B.1.351, exhibited substantial to complete escape from neutralization by monoclonal antibodies (LY-CoV555) and was granted emergency use authorization in the United States [6]. The K417N mutation of RBD could evade the neutralization of the monoclonal anti-body LY-CoV0165, which had been commercially approved [29]. As with B.1.1.7, B.1.351, P.1, and B.1.617.2 mutants, N501Y, D614G, K417N, and T478K mutations still emerged in the B.1.1.529 variant RBD, causing higher transmissibility and neutralization resistance of the B.1.1.529 variant [38].

The antibody concentration in the sixth month following the previous two doses of vaccine could not protect the human organism from infection with SARS-CoV-2 variants. Furthermore, rapid propagation of the B.1.617.2 and B.1.1.529 variants with antibodies inhibiting resistance forced many countries to consider the application of additional vaccine doses. Our research has highlighted the need for booster vaccinations when the inhibition activities of antibodies elicited by a two-dose inactivated vaccine decreased over time. Surprisingly, CoronaVac administered as a third dose significantly increased humoral immune response, thus reducing the possibility of infection by mutant strains [29] and improving protection against symptomatic illness caused by B.1.617.2 [39]. Additionally, booster vaccinations showed greater inhibitory effect on B.1.351, B.1.617.2, and B.1.1.529. The homologous vaccine could achieve a rapidly increase antibody inhibition and cross-reactive activities against the mutant strains after the booster. From a serological point of perspective, enhanced vaccine immunization can effectively solve mutant escape, possibly due to polyclonal antibodies combining with more mutation sites of SARS-CoV-2-RBD. The serum antibodies induced by the homologous booster maintained higher levels of inhibition, particularly of mutant strains, including B.1.351, B.1.617.2, and B.1.1.529, which presented strong immune escape characteristics.

Homologous and heterologous booster vaccinations with inactivated vaccines are reported to be good candidates for curbing the pandemic [39]. Our results suggested that both homologous and heterologous booster vaccination exhibit the highest efficacy against the wild-type strain and reduce the immune escape of B.1.351, B.1.617.2, and B.1.1.529 [40]. The heterologous booster vaccine elicits a stronger immune response than the homologous booster because of the different preparation routes of the primary and booster vaccines. A vaccine regimen based on the inactivated virus vaccine CoronaVac/BBIBP-CorV, which contains the whole virus with nucleoprotein, and recombinant subunit protein vaccine ZF2001, whose antigens are concentrated on RBD as a booster, can provide the immune effects of the two vaccines and exhibit more extensive inhibition against mutant strains [41] than homologous boosters. In the meantime, another study proved that the heterobooster group had higher titers of neutralizing antibodies against all Omicron subvariants than the three homologous inactivated vaccine boosters [19]. Therefore, after receiving a heterologous booster, the body should restore humoral immune responses and produce corresponding RBD-specific antibodies that are more likely to bind specifically to RBD proteins and increase inhibitory efficacy against the B.1.351, B.1.617.2, and B.1.1.529 mutant strains. More importantly, ZF2001 requires less stringent cold-chain transport and storage, which facilitates its availability in the global supply [18].

The limitations of this work were the lack of pseudovirus or real virus neutralization experiments regarding antibodies’ ability to neutralize live viruses. Numerous studies have demonstrated a close correlation between neutralization titers measured by surrogate virus neutralization tests and pseudovirus SARS-CoV-2 cultures [3,23,42,43]. In addition, our study had a small number of participants and tested samples. Participants were in the age range of 20–50, excluding special populations such as the elderly. The detection of cytokines is another important aspect of immunity. Cellular immunity data are not covered in this work.

Finally, further studies are needed to investigate the persistence of antibodies elicited by the booster vaccine and clarify whether the antibodies’ binding, inhibition, and neutralization activities toward the mutant strains sufficiently protect against them.

## 5. Conclusions

In summary, the main focus of our study was to assess the potential of vaccine-induced antibodies to inhibit the binding of hACE2 with circulating SARS-CoV-2 variants RBD. This study suggested that inhibitory antibodies elicited by homologous inactivated (BBIBP-CorV/CoronaVac) and heterologous recombinant protein subunit (ZF2001) vaccination boosters dramatically enhanced inhibitory abilities against B.1.351, B.1.617.2, and B.1.1.529 strains. Our results support the importance of enhancing immunity against epidemic strains for increasing vaccine effectiveness.

## Figures and Tables

**Table 1:** The time points and information of serum samples.

Vaccination Strategy	The First Dose	The Second Dose			Booster	
	3rd Week	1st Month	3rd Month	5th Month	1st Month	2nd Month
Inactivated Vaccine	n = 68	n = 201	n = 127	n = 68	n = 92	n = 61
ZF2001					n = 50	

Third week (range 16–27 days), 1st month (range 23–35 days), 2nd month (range 55–65 days), 3rd month (range 80–100 days), 5th month (range 145–155 days).

**Table 2:** The average inhibition rates of antibodies induced by inactivated vaccines (CoronaVac/BBIBP-CorV).

Date	Inhibition (%)					
	Wild-Type	B.1.1.7	B.1.351	P.1	B.1.617.2	B.1.1.529
1–3rd week	31.81	30.56	22.18	17.14	28.67	24.81
2–1st month	82.17	75.00	65.04	69.99	58.97	54.79
2–3rd month	61.76	51.03	41.21	45.59	40.61	35.61
2–5th month	46.25	36.28	32.23	35.48	25.64	24.58

**Table 3:** The post-booster average inhibition rates against wild-type, B.1.351, B.1.617.2 and B.1.1.529 mutant strains.

Vaccination Strategy	Inhibition (%) ± SD			
	Wild-Type	B.1.351	B.1.617.2	B.1.1.529
Inactivated Booster—1 month	88.30 ± 9.05	79.01 ± 12.32	76.34 ± 12.07	72.07 ± 12.88
ZF2001 Booster—1 month	92.28 ± 4.62	85.31 ± 8.15	82.10 ± 7.36	81.27 ± 9.30
Inactivated Booster—2 month	80.74 ± 10.35	73.24 ± 15.71	69.12 ± 12.61	71.54 ± 12.30

## Data Availability

Data are contained within the article.

## Supplementary Materials

The following supporting information can be downloaded at: https://www.mdpi.com/article/10.3390/vaccines10122110/s1, Figure S1: SDS-PAGE of SARS-CoV-2 wild-type and mutant strains spiking proteins’ RBD recombination proteins and hACE2 recombination protein; Figure S2: Enzyme-linked immunoassay competition test determined the negative cutoff value from testing over negative (n = 89) human serum against all variants (wild-type, B.1.1.7, B.1.351, P.1, B.1.617.2, and B.1.1.529); Figure S3: The average inhibition rates of the serum samples of 32 volunteers to wild-type and mutant strains at each time point were analyzed and the p values were presented; Figure S4: Inhibition curves of each of the serum from volunteers (n = 5) with receiving the two primary inactivated vaccine were shown; Figure S5: Inhibition curves of each of the serum from volunteers (n = 5) on the first month after receiving booster vaccine (CoronaVac/BBIBP-CorV) and ZF2001 are shown; Table S1: Differences of inhibition rates of primary inactivated (CoronaVac/BBIBP-CorV) vaccine; Table S2: The average inhibition rates of serum samples of 32 volunteers collected at different time intervals against wild-type and mutant strains B.1.1.7, B.1.351, P.1, B.1.617.2, and B.1.1.529; Table S3: Differences of inhibition rates of inhibition of booster vaccine; Table S4: Inhibiting activity of the two primary vaccine-induced against wild-type, B.1.1.7, B.1.351, P.1, B.1.617.2, and B.1.1.529; Table S5: Inhibiting activity of the homologous inactivated booster or heterologous (ZF2001) booster vaccine-induced against wild-type, B.1.351, B.1.617.2 and B.1.1.529.
