# Supplementary material for: Antibodies Induced by Homologous or Heterologous Inactivated (CoronaVac/BBIBP-CorV) and Recombinant Protein Subunit Vaccines (ZF2001) Dramatically Enhanced Inhibitory Abilities against B.1.351, B.1.617.2, and B.1.1.529 Variants
Source: Vaccines (Basel). 2022 Dec 9;10(12):2110. doi: 10.3390/vaccines10122110 (PMC9782763; doi:10.3390/vaccines10122110)
Supplement: Supplementary file 1 [file vaccines-10-02110-s001.zip › vaccines-2079077-supplementary.pdf]

Supplementary information

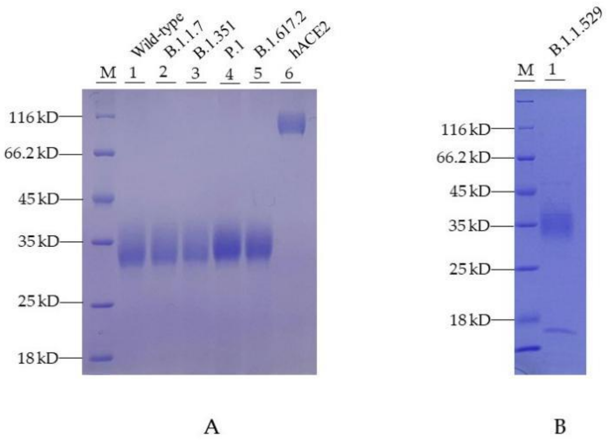

Figure S1. SDS-PAGE of SARS-CoV-2 wild-type and mutant strains spiking proteins' RBD recombination proteins and hACE2 recombination protein.

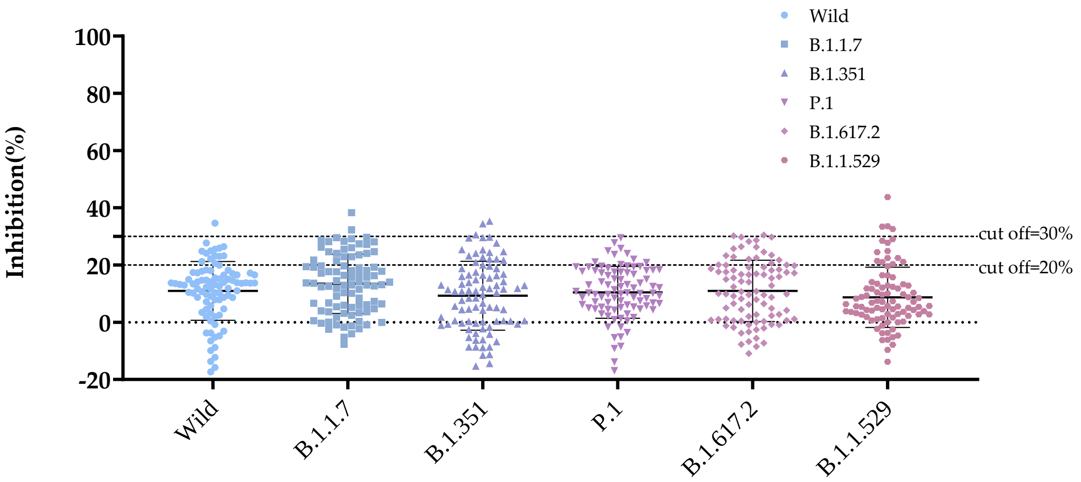

Figure S2. Enzyme-linked immunoassay competition test determined the negative cutoff value from testing over negative (n=89) human serum against all variants (wild-type, B.1.1.7, B.1.351, P.1, B.1.617.2, and B.1.1.529). Dashed lines indicated 20% or 30% cut-off. As the negative cutoff value was 30%, competition test specificity against wild-type, B.1.1.7, B.1.351, P.1, B.1.617.2, and B.1.1.529 were 98.88%, 97.75%, 96.63%, 100.00%, 97.75%, 95.51%, respectively.

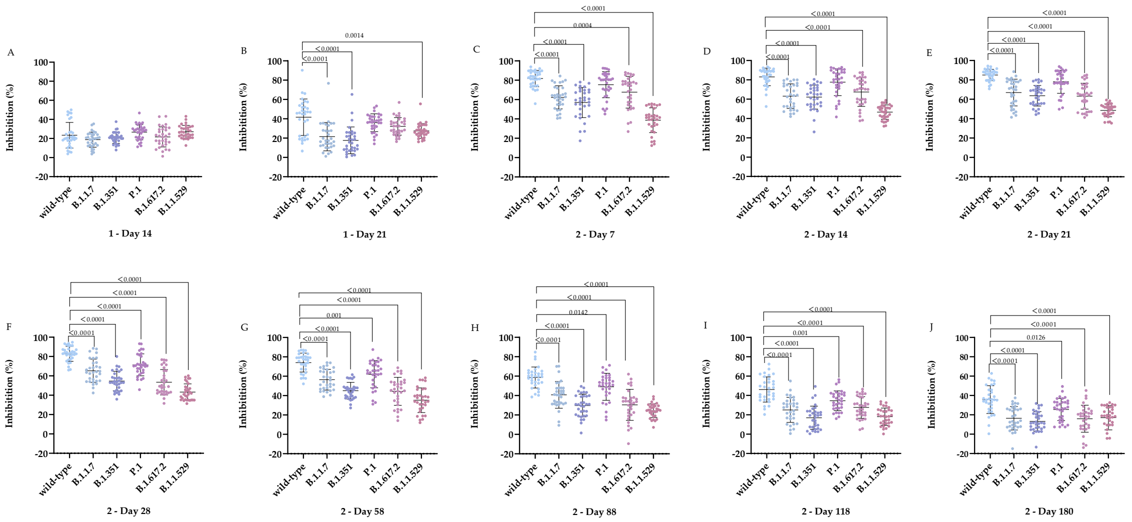

Figure S3. The average inhibition rates of the serum samples of 32 volunteers to wild-type and mutant strains at each time point were analyzed and the P values were presented. Specific antibodies in vaccinated serum demonstrate inhibitory abilities for wild-type and mutant strains at the same time point. (A) 1-Day14. (B) 1-Day21. (C) 2-Day7. (D) 2-Day14. (E) 2-Day21. (F) 2-Day28. (G) 2-Day58. (H) 2-Day88. (I) 1-Day118. (J) 1-Day180.

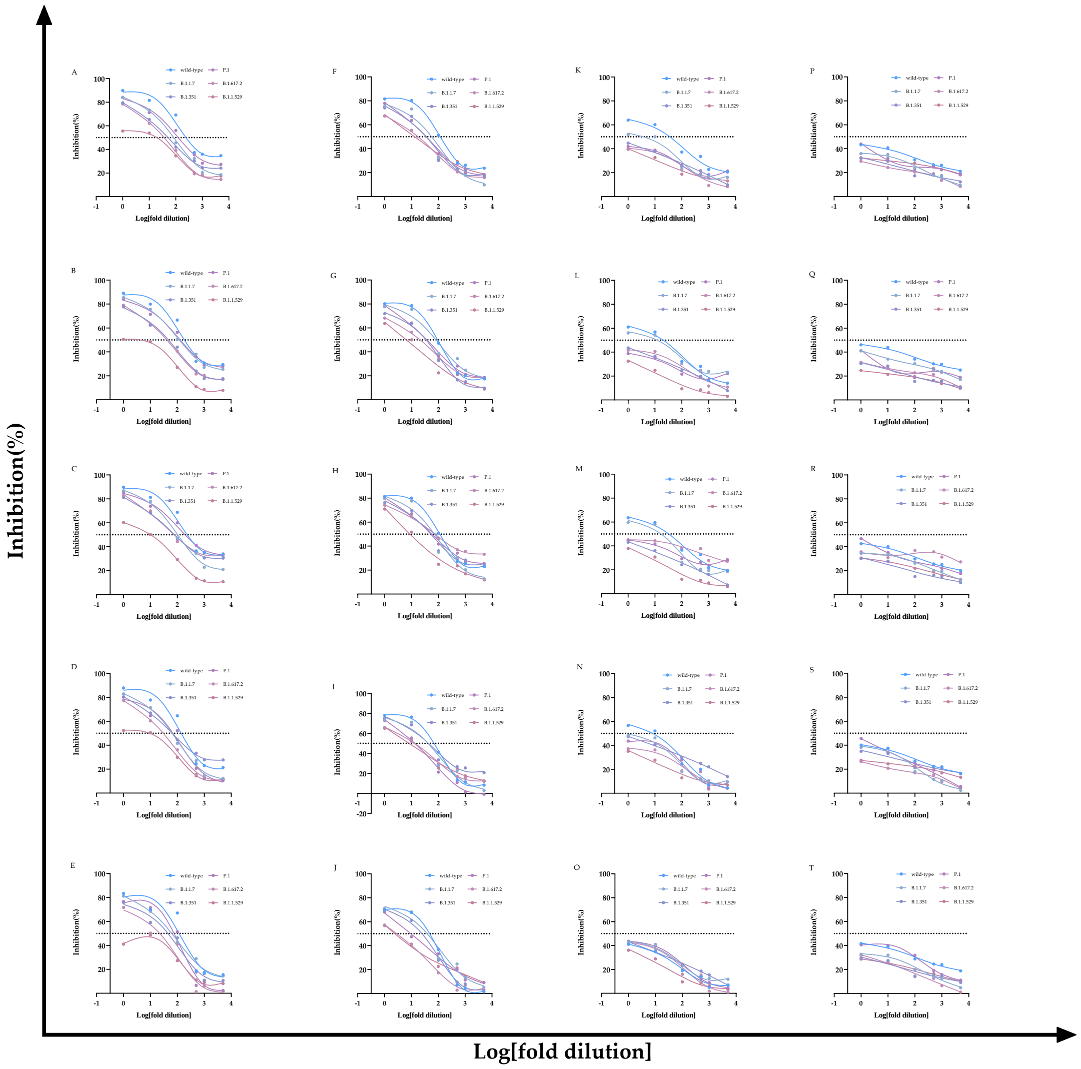

Figure S4. Inhibition curves of each of the serum from volunteers (n=5) with receiving the two primary inactivated vaccine were shown. A-E: serum collected on the 1st month after two primary inactivated vaccine, F-J: serum collected on the 2nd month after two primary inactivated vaccine, K-O: serum collected on the 3rd month after two primary inactivated vaccine, P-T: serum collected on the 5th month after two primary inactivated vaccine.

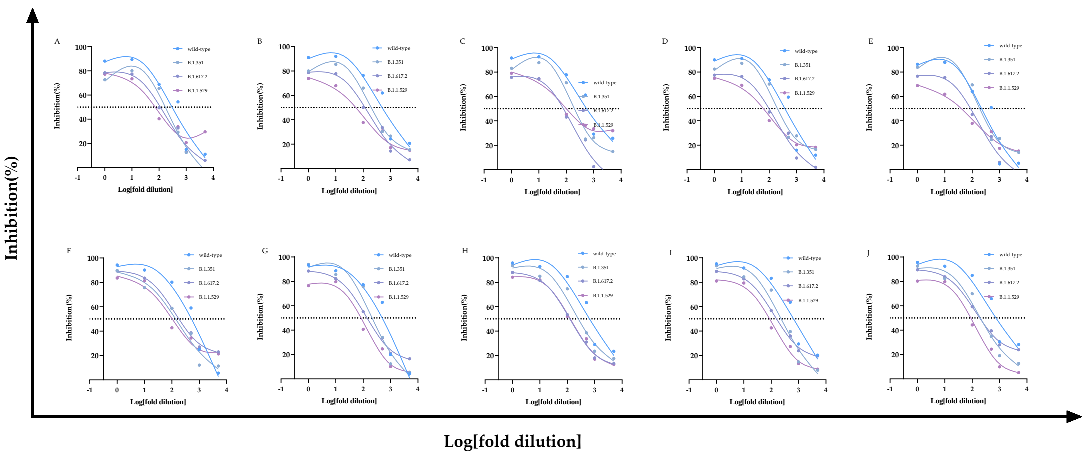

Figure S5. Inhibition curves of each of the serum from volunteers (n=5) on the first month after receiving booster vaccine (CoronaVac/BBIBP-CorV) and ZF2001 are shown. A-E: CoronaVac/BBIBP-CorV booster, F-J: ZF2001.

Table S1. Differences of inhibition rates of primary inactivated (CoronaVac/BBIBP-CorV) vaccine.

	**1 – 3rd week**		**2 – 1st month**		**2 – 3rd month**		**2 – 5th month**	
**wild-type vs. B.1.1.7**	ns	0.985	****	<0.0001	****	<0.0001	***	0.0001
**wild-type vs. B.1.351**	****	<0.0001	****	<0.0001	****	<0.0001	****	<0.0001
**wild-type vs. P.1**	****	<0.0001	****	<0.0001	****	<0.0001	****	<0.0001
**wild-type vs. B.1.617.2**	ns	0.542	****	<0.0001	****	<0.0001	****	<0.0001
**wild-type vs. B.1.1.529**	**	0.0013	****	<0.0001	****	<0.0001	****	<0.0001
**B.1.1.7 vs. B.1.351**	****	<0.0001	****	<0.0001	****	<0.0001	ns	0.3604
**B.1.1.7 vs. P.1**	****	<0.0001	**	0.008	**	0.0025	ns	0.9993
**B.1.1.7 vs. B.1.617.2**	ns	0.9066	****	<0.0001	****	<0.0001	****	<0.0001
**B.1.1.7 vs. B.1.1.529**	*	0.0127	****	<0.0001	****	<0.0001	****	<0.0001
**B.1.351 vs. P.1**	*	0.0314	**	0.0058	*	0.0474	ns	0.653
**B.1.351 vs. B.1.617.2**	**	0.0033	***	0.0003	ns	0.998	*	0.023

**B.1.351 vs. B.1.1.529**	ns	0.5641	****	<0.0001	***	0.0003	***	0.0003
**P.1 vs. B.1.617.2**	****	<0.0001	****	<0.0001	**	0.0099	***	0.0005
**P.1 vs. B.1.1.529**	****	<0.0001	****	<0.0001	****	<0.0001	****	<0.0001
**B.1.617.2 vs. B.1.1.529**	ns	0.216	*	0.0189	***	0.0009	ns	0.9937

NS = not significant

Table S2. The average inhibition rates of serum samples of 32 volunteers collected at different time intervals against wild-type and mutant strains B.1.1.7, B.1.351, P.1, B.1.617.2, and B.1.1.529.

Date	Inhibition (%)					
	wild-type	B.1.1.7	B.1.351	P.1	B.1.617.2	B.1.1.529
**1-Day 14**	23.41	18.76	20.78	26.65	21.67	27.07
**1- Day 21**	41.63	21.54	17.70	35.98	32.11	27.42
**2- Day 7**	81.71	62.29	56.91	75.32	67.63	38.65
**2- Day 14**	83.04	63.14	62.17	77.64	67.45	46.62
**2- Day 21**	85.02	66.95	63.62	78.09	63.21	48.52
**2- Day 28**	82.74	65.21	54.67	71.38	53.42	43.26
**2- Day 58**	74.09	56.46	45.01	62.10	44.41	35.22
**2- Day 88**	58.61	40.71	29.78	49.05	30.45	24.49
**2- Day 118**	46.13	25.10	17.00	34.55	27.61	18.06
**2- Day 180**	35.71	16.45	13.23	25.59	15.97	17.08

Table S3. Differences of inhibition rates of inhibition of booster vaccine.

	Inactivated Booster -1st month		ZF2001 Booster -1st month		Inactivated Booster-2st month	
wild-type vs. B.1.351	****	<0.0001	****	<0.0001	*	0.0127
wild-type vs. B.1.617.2	****	<0.0001	****	<0.0001	****	<0.0001
wild-type vs. B.1.1.529	****	<0.0001	****	<0.0001	****	<0.0001
B.1.351 vs. B.1.617.2	ns	0.4473	ns	0.1729	ns	0.384
B.1.351 vs. B.1.1.529	*	0.0119	ns	0.1561	ns	0.2843
B.1.617.2 vs. B.1.1.529	ns	0.3384	ns	0.991	ns	0.9972

NS = not significant

Table S4. Inhibiting activity of the two primary vaccine-induced against wild-type, B.1.1.7, B.1.351, P.1, B.1.617.2, and B.1.1.529.

Date/volunteers	IC50					
the 1st month	wild-type	B.1.1.7	B.1.351	P.1	B.1.617.2	B.1.1.529
1#	358.90	75.41	51.08	130.60	28.50	5.64
2#	228.20	129.40	34.92	138.30	33.80	2.47
3#	334.00	103.40	97.52	254.10	114.80	5.52
4#	137.90	49.94	71.78	43.46	21.85	3.25
5#	89.38	48.43	25.35	41.36	10.81	0.99
the 2nd month	wild-type	B.1.1.7	B.1.351	P.1	B.1.617.2	B.1.1.529
6#	107.50	36.83	26.50	32.36	12.30	13.85
7#	69.70	62.99	19.43	34.25	15.02	6.26

8#	100.20	53.74	48.02	63.23	86.58	10.51
9#	42.18	24.69	36.14	9.90	9.07	8.16
10#	26.33	25.32	15.18	8.95	3.09	2.33
the 3rd month	wild-type	B.1.1.7	B.1.351	P.1	B.1.617.2	B.1.1.529
11#	22.74	3.49	0.59	0.35	0.52	0.12
12#	12.20	7.10	0.50	0.07	0.64	0.12
13#	20.55	9.82	0.48	0.29	0.56	0.26
14#	5.95	2.20	0.88	1.56	0.40	0.11
15#	0.59	0.86	0.46	1.24	1.12	0.26
the 5th month	wild-type	B.1.1.7	B.1.351	P.1	B.1.617.2	B.1.1.529
16#	/	/	/	/	/	/
17#	/	/	/	/	/	/
18#	/	/	/	/	/	/
19#	/	/	/	/	/	/
20#	/	/	/	/	/	/

IC50 indicates inhibition concentration 50%. IC50 and values were determined using GraphPad Prism 8.0.2 software. /: Not matching result with IC_{50}.

Table S5. Inhibiting activity of the homologous inactivated booster or heterologous (ZF2001) booster vaccine-induced against wild-type, B.1.351, B.1.617.2 and B.1.1.529.

		IC50			
the 1st month after booster dose		wild-type	B.1.351	B.1.617.2	B.1.1.529
	1#	292.7	130.7	83.36	71.03

CoronaVac/BBIBP-CorV booster	2#	516.9	201.4	88.91	39.69
	3#	618.7	216.1	50.09	123.7
	4#	368.4	220.6	68.93	48.11
	5#	203.9	204.0	58.30	26.92
ZF2001 booster	6#	450.4	122.6	207.3	106.7
	7#	411.70	211.60	141.30	61.06
	8#	686.8	278.8	120.0	118.6
	9#	649.00	225.80	169.10	70.53
	10#	800.1	201.6	221.9	69.13

IC50 indicates inhibition concentration 50%. IC50 and values were determined using GraphPad Prism 8.0.2 software.
